# A Bioinformatics Pipeline for the Analyses of Viral Escape Dynamics and Host Immune Responses during an Infection

**DOI:** 10.1155/2014/264519

**Published:** 2014-06-10

**Authors:** Preston Leung, Rowena Bull, Andrew Lloyd, Fabio Luciani

**Affiliations:** Inflammation and Infection Research Centre, School of Medical Sciences, The University of New South Wales, Sydney, NSW 2052, Australia

## Abstract

Rapidly mutating viruses, such as hepatitis C virus (HCV) and HIV, have adopted evolutionary strategies that allow escape from the host immune response via genomic mutations. Recent advances in high-throughput sequencing are reshaping the field of immuno-virology of viral infections, as these allow fast and cheap generation of genomic data. However, due to the large volumes of data generated, a thorough understanding of the biological and immunological significance of such information is often difficult. This paper proposes a pipeline that allows visualization and statistical analysis of viral mutations that are associated with immune escape. Taking next generation sequencing data from longitudinal analysis of HCV viral genomes during a single HCV infection, along with antigen specific T-cell responses detected from the same subject, we demonstrate the applicability of these tools in the context of primary HCV infection. We provide a statistical and visual explanation of the relationship between cooccurring mutations on the viral genome and the parallel adaptive immune response against HCV.

## 1. Introduction

The complete lack or limited efficacy of vaccines for rapidly mutating viruses causing chronic infections (e.g., HIV and HCV) or seasonal pandemics (Influenza) is due to the extreme and rapid adaptation dynamics of these viruses at both the within and between host level. The extremely high mutation rate of these genomes results in single nucleotide polymorphisms (SNPs) emerging in the viral genome in a short time scale, which in turn gives rise to immune escape phenotypes. These mutations result in new viral variants that avoid detection from the adaptive immune responses (T-cell and B-cell responses), which previously targeted the original virus. However, these mutations are likely to have fitness costs and limit the successful transmission of viral escape variants to a new host. Viruses have to compensate for these costs with additional mutations. Therefore, to understand viral immune escape it is important to study the cooccurrence of multiple mutations on the same genome, as these are the source of compensatory mutations that can drive successful transmission of immune escape variants at the population level. An important lesson learned from the failed T-cell based HIV vaccine (STEP) [1] trials is that T-cell induced responses are not protective against the virus if their targets are viral antigens with a high likelihood of beneficial compensatory mutations, which would allow rapid and successful immune escape. Rather, the data suggests that successful vaccines need to induce strong immune responses against epitopes for which viral escape variants are unlikely to establish a successful new viral population. For instance, T-cell responses should be induced on viral epitopes that are associated with significant deleterious effects on virus viability.

### 1.1. Next Generation Sequencing Technology (NGS)

Recent advances in high-throughput sequencing allow researchers to generate very large data sets of pathogen genomes as well as that of host genomes and transcriptomes. These data, although useful, also carry a complexity that requires computational analyses to understand and represent the biological phenomena. NGS, in particular, has become a powerful tool for deep sequencing analyses of highly variable genomic populations, such as those arising during an infection with rapidly mutating pathogens, or metagenomics [2]. Similarly, in immunology, NGS is increasingly utilized for sequencing of highly polymorphic protein encoding regions, which are key elements of pathogen recognition, including HLA genes, T-cell receptor [3–5], and the B cell receptors [6]. NGS technology has been also utilized to study the evolution of complex viral populations evolving during an infection within host, such as the detection of rare viral variants in hepatitis C virus (HCV) [7], or in HIV [8]. These data have a strong significance in the study of the dynamics of immune escape as it provides deep insights into the kinetics and extent of viral immune escape [9].

### 1.2. HCV and Host Immune Response

HCV establishes chronic infections in 60–80% of the infected population [10]. Those individuals who have chronic infection outcomes can eventually undergo liver damage and the development of hepatocellular carcinoma (HCC) [11], the fifth most common cancer worldwide [12]. HCV is a single stranded RNA virus; it evolves by rapidly mutating its genome during a single infection with an estimated mutation rate of 1.2 × 10^−4^ per site per cell [13]. Similar to other RNA viruses, HCV mutates frequently across the genome, resulting in a high degree of heterogeneity between the seven different HCV genotypes, which carry only ~65% identity. The high mutation rate occurs because the viral encoded RNA-dependent RNA polymerase lacks proof-reading capacity, resulting in at least one error in each genome copied [14]. Consequently, HCV exists as a diverse and evolving population within each infected host. However, it is estimated that about a third of the mutations introduced are deleterious [13], hence many new variants are eliminated in a potent negative selection process [15]. These high error rates are also advantageous to the virus, as they drive rapid adaptation to changing environmental landscapes, such as transmission to a new host or an emerging immune escape variant [16, 17]. Therefore, there is a tradeoff between beneficial and deleterious mutants. This rapid evolution is critical for the survival of these viruses during the establishment and maintenance of chronic viral infections. It facilitates viral escape from host immune responses [18] and optimizes replication efficiency.

Whether infected individuals clear HCV or have viral persistence can be determined by the host immune response [9, 19]. In particular, observation of HCV cytotoxic T-cells (expressing CD8+ marker, CD8+ T-cells henceforth) has shown that these cells contribute to the infection's outcome, where virus-specific CD8+ T-cells are crucial in controlling HCV replication [18, 19]. Although HCV specific CD8+ T-cells are found in acute HCV infection, their efficacy in persistent infection is limited by several factors including T-cell exhaustion and immune escape. The drive behind immune escape is due to the error prone replication mechanism of HCV, thus allowing adaptation to the immune selection pressure through genomic sequence mutations [18]. For example, nonsynonymous mutations in the viral genomic sequence can lead to a peptide change at the protein level of the genome. This mutation can abrogate the immune recognition pathway at several levels. For instance, this mutation can impair antigen processing from viral proteins, as well as the presentation of CD8+ T-cell epitope [20].

### 1.3. Transmitted/Founder Virus

The transmitted/founder virus is the strain that successfully infects a new host after a transmission event. In HCV studies it has been shown that very few (1–3) transmitted/founder viruses are present in acute infections [7]. An additional study has shown that a genetic bottleneck (an event where genetic variation is greatly reduced) occurs later in infection when selective pressure from the host immune response acts against the transmitted/founder virus [7]. The study observed that as a result of genetic bottleneck events, new viral populations emerge in subjects that become chronically infected with HCV. These arising variants were characterized by fixation events, namely, mutations occurring in >90% of circulating viral genomes. Close examination on viral sequences found only a minority of these fixations events were likely to be under immune-driven selection.

### 1.4. Cooccurrence of Genomic Mutations and Its Impact on the Dynamics of Immune Escape

Most genomic mutations in RNA viruses are deleterious [13] and can lead to the extinction of the viral variant through negative selection. A smaller proportion of these mutations may have beneficial effects on the survival of the virus. For instance, cooccurring mutations where a new mutation can compensate for the fitness loss given by previously generated mutations, termed compensatory mutations, are beneficial to the survival of the virus [21]. Thus cooccurring mutations can lead the virus to immune escape or even drug resistance phenotypes. As a consequence, it is crucial to study the interactions between immune responses and the resulting mutations to better understand the mechanism behind viral escape.

### 1.5. Visualizing Genomic and Immunological Data

Given the level of complexity involved with HCV and the increasingly vast amount of generated data from technologies like NGS, representation of the virus's genomic information is becoming increasingly difficult. As a result, computational methods are becoming more and more crucial in terms of data processing, analysis, visualization, and ultimately understanding biological information. The need for software packages allowing combined analysis of viral evolution, detection of compensatory mutations, and identification of immune escape patterns is evident. Thus the goal of this study was to develop a set of computational and statistical tools to analyze immune escape viral variants. We address this issue by taking the dynamic HCV genome as an example and applying the viral sequence data to our bioinformatics pipeline.

## 2. Materials and Methods

### 2.1. Data

#### 2.1.1. NGS Viral Sequences

Longitudinal viraemic samples were collected and sequenced from a subject (Ch_240) that developed chronic HCV infection from genotype 3a. The longitudinal samples were deep sequenced using NGS 454 Roche (for more details see Bull et al. [7]). Four viraemic samples from Ch_240 were deep sequenced, corresponding to 44 days after infection (DPI), 57 DPI, 220 DPI, and 538 DPI, respectively. 454 Roche data were retrieved in a pair of fasta (*.fna*) and quality score (*.qual*) format file from each individual time point.

#### 2.1.2. CD8+ T-Cell Responses

HCV specific CD8+ T-cell response data were available for Ch_240. These responses were measures of HCV specific CD8+ T-cell responses using ELISPOT assay. This assay detected active T-cell responses against specific epitopes, a short amino acid sequence recognized by CD8+ T-cells via presentation of HLA-epitope complex on the surface of infected cells. Two CD8+ T-cell responses specific for epitopes _1602_RAQAPPPSW_1610_ and _1633_RLGPVQNEV_1641_ were utilized in this study. These epitopes are both located in the NS3 region of the HCV genome (nucleotide region 4000–5499 and amino acid region 1200–1800, according to the HCV gt1 reference genome H77, GenBank accession number AF009606).

#### 2.1.3. Single Genome Amplification (SGA) Sequences

Longitudinal sequence data were retrieved from publicly available data [22]. We analyzed sequences from three time points within the first 4 weeks of acute infection from one subject (10012) infected with HCV genotype 1a.

### 2.2. Tools for Computational Analysis

#### 2.2.1. Quality Control and Sequence

Each fasta and quality score file are processed by* choplqb.py* with options* −w 8 −t 15* for initial data cleaning. Refined outputs from* choplqb.py* are then given to qualfa2fq.pl to combine the fasta file and quality score file into a single fastq file. This is then used for sequence alignment using the Burrow Wheel algorithm, implemented in the software package BWA [23]. BWA and qualfa2fq.pl are both available at http://sourceforge.net/projects/bio-bwa/files/ (see* README.md* at the GitHub repository located below for more information).

#### 2.2.2. HCV NS3 Reconstruction

Refined NGS data are processed by SamTools [24] (version 0.1.19, http://sourceforge.net/projects/samtools/files/latest/download) to convert the sam file into bam format in order to apply the data to the genome reconstruction software ShoRAH [25] (Short Reads into Assembly Haplotypes). This software allows for reconstruction of partial or full genome sequences from a mixed population of genomes, henceforth reconstruction of viral haplotypes (this software is available at http://www.bsse.ethz.ch/cbg/software/shorah). The options for ShoRAH include* −a 0.05 −w 300* and a length restriction of 1500 nucleotides in the NS3 region 4000–5499. Sets of reconstructed and aligned viral sequence files in fasta format are retrieved using this software. For this analysis, only full-length (1500 nt) reconstructed fasta files (*.popl*) from ShoRAH that carry viral variants with frequency of occurrence of 1% or greater are applied to this pipeline.

#### 2.2.3. Reducing Insertion and Deletion Errors

Viral sequences reconstructedfor each time point are piped into* indelRemover.py* using option* −fq 2* for insertion and deletion reduction. The output is another fasta format file with reduced insertion and deletions around homopolymers regions in the viral sequences.

#### 2.2.4. Analysis of Cooccurring Mutations

Fasta files of viral genome populations are given to* a-smupfi.py* with options* −g −gf 0.01 −e −sc 242 −m 1-2 −s 4000–5499,* which produced four text files containing the combinations of shared mutations and their frequency of occurrence between viral sequences observed in the data set. From these four files, the file with suffix* EasyOutputShared.txt* is used as input data for* javssim.py* with option* −e*. The result provided by* javssim.py* contains a list of shared combinations of mutations. This tool utilizes the Jaccard similarity coefficient, calculated for a given pair of mutations *A* and *B*, as *J* = *N*_*AB*_/(*N*_*AB*_ + *N*_*A*0_ + *N*_0*B*_), where *N*_*AB*_ represents the number of variants carrying mutations *A* and *B*, *N*_*A*0_ represents the number of variants containing *A* but not *B*, and *N*_0*B*_ represents the number of variants containing *B* but not *A*. The algorithm implemented here for the detection of statistically significant pairs was taken from Rhee et al. [26]. Briefly, statistically significant pairs are identified from the expected Jaccard similarity coefficient and its standard error assuming the two mutations independently distributed. *J*_RAND_ was calculated as the mean Jaccard similarity coefficient after 2,000 random rearrangements of the *X* or *Y* vector (containing 0 or 1 for presence or absence of a mutation, resp.). *J*_SE_ was calculated using a jackknifed procedure, which removed one sequence at a time, repeatedly for each sequence. The standardized score *Z*, *Z* = (*J* − *J*_RAND_)/*J*_SE_, indicates a significant positive association (*Z* > 1.65) or a significant negative association (*Z* < −1.65) at an unadjusted *P* < 0.05.

The results from this analysis are then found in the file with suffix* EasyOutput.txt* from* javssim.py*. This file without a header can be parsed into* jaccardToCircos.py* with option* −li* for format conversion into a style that can be understood by Circos [27] (software is available at http://circos.ca/) to draw connections between significant cooccurring mutations.

#### 2.2.5. Analysis of Covariance

The covariance analysis was performed on viral sequences translated into amino acid using ExPASy tools [28]. These are then inputted into* omes.py* for calculation of the covariance score, according to the observed minus expected squared (OMES) method [29] as

(1) $$\begin{array}{c} \text{Co}{\text{v}}_{\text{score}}=\frac{\sum_{1}^{L}{\left(N_{\text{obs}}-N_{\exp}\right)}^{2}}{N_{\text{sequences}}},\\ N_{\exp}=\frac{\left(F_{aj}\times F_{bk}\right)}{N_{\text{sequences}}}, \end{array}$$

where each paired position *j* and *k* has residue *a* and residue *b*, respectively. *F*_*aj*_ is the frequency of occurrence of *a* at position *j* and *F*_*bk*_ is the frequency of occurrence of *b* at position *k*. *N*_sequences_ is the number of sequences at positions *j* and *k*. *N*_obs_ is the frequency of occurrence of the pair *a* and *b* at positions *j* and *k*. *L* is the number of unique pairs counted at positions *j* and *k*. This output is parsed into* sifconvertor.py* to generate a network file (*.sif*), which can be parsed into Cytoscape [30] (software is available at http://www.cytoscape.org/) for simple network visualization. For this analysis only mutation pairs that have a covariance score equal to or greater than 0.5 (cut-off value taken from Aurora et al. [31]) were considered for further analysis.

#### 2.2.6. Histogram Generation

Using the same set of output from* a-smupfi.py*, the file with suffix* EasyOutputUnique.txt* is applied as input into* circosconverter.py* with options* −hi* to generate Circos format histograms. The bam file from the NGS data (also used for generating haplotypes with ShoRAH) is used to identify SNPs within the viral population. This SNP detection was performed using LoFreq (version 0.5.0) [32] (software is available at http://sourceforge.net/projects/lofreq/files/) using default options for additional SNP calling. However, other software can be utilized for SNP detection from NGS bam file. SNPs data are then parsed into* snpExtractor.py* and then those outputs are given to* snpExtractConvertor.py* for Circos format conversion into histogram files.

#### 2.2.7. Phylogenetic Tree Generation

Viral sequences of all four time points from Ch_240 have been appended into one sequence file and given as input to PhyML Ver. 3.0.1 [33] (software available at http://www.atgc-montpellier.fr/phyml/) changing options* S* (Tree topology search operations) to* Best of NNI and SPR (extensive tree search)* and* B* (nonparametric bootstrap analysis) to* 1000*. The phylogenetic tree was generated under the substitution model HKY85 using estimated *γ* distribution on sites. The output file from PhyML is represented and visually edited through the software FigTree Ver. 1.3.1 (software available at http://tree.bio.ed.ac.uk/software/figtree/).

Script names in italics are original tools and are available at the GitHub repository: https://github.com/PrestonLeung/SMuPFi-Repository.

## 3. Results

### 3.1. An Overview of the Pipeline

**Figure 1:**
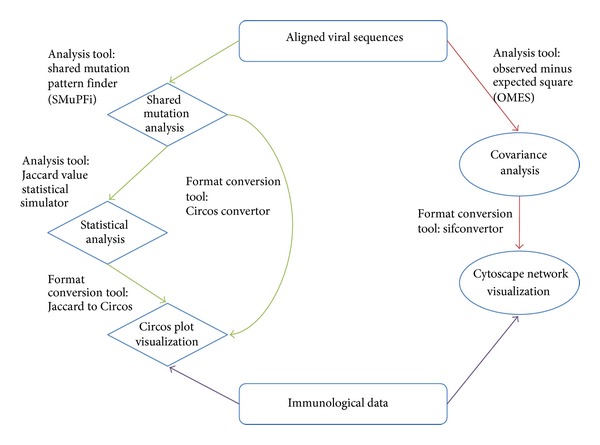
Flowchart representing the bioinformatics pipeline. Shared Mutation Analysis workflow is indicated by green arrows. Covariance Analysis workflow is indicated by red arrows. Both branches of the pipeline require an aligned viral sequence file in fasta format. Immunological data are optional and can be both experimental or bioinformatics predicted.

A workflow of the proposed pipeline for the analysis of genomic and immunological data related to immune escape during HCV infection is shown in Figure 1. The pipeline builds a path that connects NGS data, viral sequence quality control, alignment software, haplotype reconstruction (e.g., using ShoRAH), and identification of the distribution of variants sharing a number of SNPs. It also provides graphical representation of these findings through Circos and Cytoscape. In this pipeline, we present the Shared Mutation Pattern Finder (SMuPFi), a novel algorithm package that analyses cooccurring mutation patterns with a series of supporting tools inside the package. Figure 1 shows the workflow that describes the cooccurring mutation analysis and the covariance network analysis. The pipeline uses viral sequences and immunological data as the input source where the region of interest is selected based on immunological data. The cooccurring mutation analysis uses viral sequences as the main data source. Combinations of mutation with length of 2 and occurring on the same viral genome are then selected. Combinations of mutation pairs that are shared by more than two viral variants are identified for further analysis. This dataset is then used to identify those combinations of coupled mutations that occur in the viral population at a frequency higher than that expected by random mutation. These are recognized through a test for statistical significance implementing the Jaccard similarity coefficient (see Section 2). Statistically significant cooccurring mutations are identified from the expected distribution of cooccurrence assuming independency of the two mutations of interest. These pairs are then graphically represented via Circos plots. The covariance network analysis similarly uses viral sequences as input data. It identifies pairs of mutated sites and utilizes the paired positions and frequencies to calculate a covariance score. This score reveals those pairs that are likely to have a relationship such that their cooccurrence is more than an expected value, which is calculated as the product of frequencies of each residue in the pair, divided by the total number of sequences (OMES see Section 2). The data is then represented in a network using Cytoscape.

### 3.2. Application of the Pipeline to HCV

Virological and immunological data from a subject (Ch_240) chronically infected with HCV has been applied to the pipeline using deep sequencing data available from four time points (44 DPI, 57 DPI, 220 DPI, and 538 DPI). The input data consisted of viral sequences from a segment of the NS3 region (nucleotide region 4000–5499 and amino acid region 1200–1800, see Section 2 for more details) and immunological data, which were available ELISPOT measurements of CD8+ T-cell responses specific for two HCV antigenic epitopes _1633_RLGPVQNEV_1641_ and _1602_RAQAPPPSW_1610_. Both of these epitopes were located within the sequenced NS3 region.

**Figure 2:**
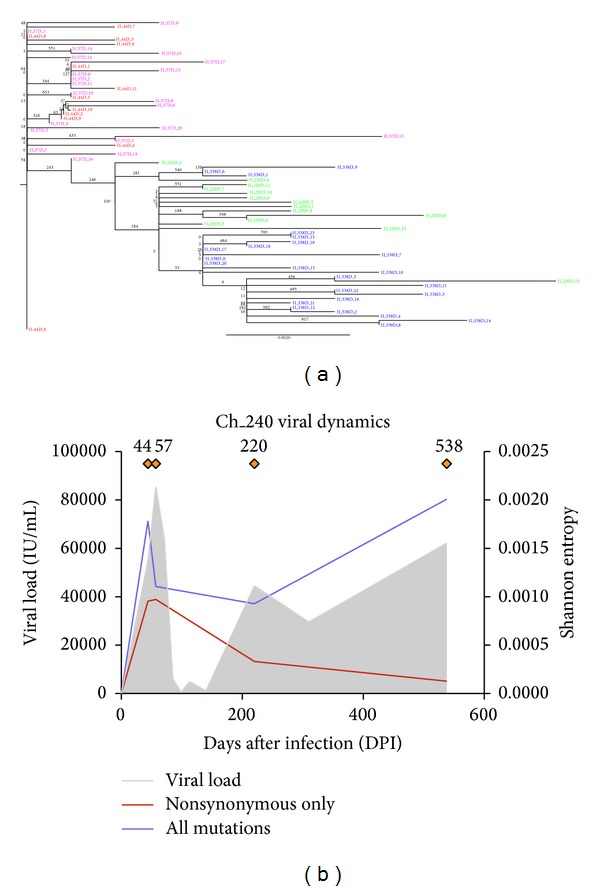
Phylogenetic tree representation of viral sequences obtained from Ch_240. Phylogenetic tree of circulating HCV variants from sequences derived from the NS3 region (nucleotide region 4000–5499, amino acid region 1200–1800). This tree shows the significant rapid evolution of HCV genome over the course of the infection from acute phase (in red viral sequences from 44 DPI, in pink those for 57 DPI) through the chronic phase of infection (in green sequences from 220 DPI, and in blue from 538 DPI). The infection is characterized by two sequential genetic bottlenecks, one soon after the acute phase of infection, the other after 220 DPI, both indicating substantial changes in the circulating viral populations. The label H_44D_0 indicates the transmitted/founder virus used to root the phylogenetic tree. The numbers at branches indicate the bootstrap value after resampling 1000 times. Viral Dynamics plot (b) shows the viral load (grey shading) over time, while the orange diamonds indicate available deep sequence data (from left to right: 44, 57, 220, 538 DPI). Shannon entropy calculated from the full distribution of HCV genomic mutations and the one from the distribution of nonsynonymous mutations only are indicated by a purple line and red line, respectively.

Figure 2 shows a phylogenetic tree representation of the viral sequences from the partial NS3 region of the HCV genome of subject Ch_240 displaying the genetic distances measured by nucleotide differences using the transmitted/founder virus (labeled H_44 DPI_0) as the root of the tree. In this subject it is evident that the viral population significantly evolved from the transmitted/founder virus during the early acute phase of the infection with rapid genomic diversification undergoing sequential genetic bottlenecks events. In this figure, the viral dynamics plot (zoomed panel in Figure 2) describes the level of variability in the genome using Shannon entropy measured across the full HCV genome. This plot shows the first genetic bottleneck occurring at approximately 100 DPI where viral diversity significantly decreases, along with the viral load and the loss of the dominant viral variants [7]. At 200 DPI, the viral load increases, along with the number of distinct circulating NS3 variants, thus indicating a new viral population characterizing the chronic phase of infection. Of note, a second genetic bottleneck occurs between 220 and 538 DPI. While the figure shows sequences from 44 DPI and 57 DPI evolving into the latter group (220 DPI and then 538 DPI), notably there is a large time period of approximately 300 days between 220 DPI and 538 DPI. This rapid evolution is a major component driving the success of the virus to establish chronic infection and escape the host immune response. These mutations are further explored in the following analyses.

### 3.3. Identification of Cooccurring Genomic Mutations

**Figure 3:**
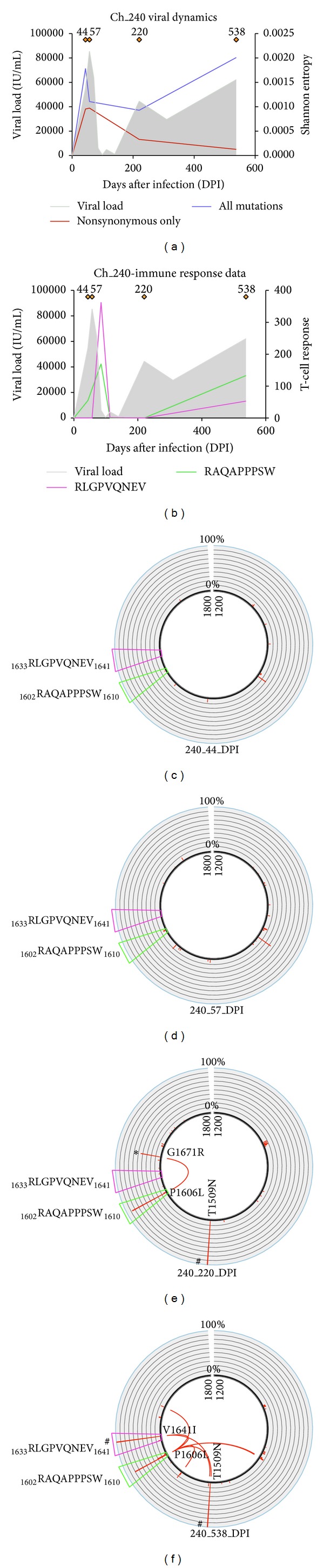
A representative example of the output of the pipeline applied on longitudinally collected viral sequences and immune responses data from a patient chronically infected with HCV. (a) The same viral dynamics plot carried over from Figure 2. (b) The experimental results from ELISPOT detailing the measurement of two HCV specific CD8+ T-cell responses targeting epitopes _1602_RAQAPPPSW_1610_ (green) and _1633_RLGPVQNEV_1641_ (pink). Immune responses are first detected at around 50 DPI at low amount. These responses then increase in magnitude at 80–100 DPI and then decline over the course of the infection. (c–f) Circos plots representing the partial NS3 region (amino acid region 1200–1800) in Ch_240 that contain the two epitopes targeted by the CD8+ T-cell responses in (b). The red histogram around the circle shows the frequency of occurrence of nonsynonymous mutations with a scale from 0% to 100% at that site. These two CD8+ T-Cell epitopes _1602_RAQAPPPSW_1610_ (green trapezium) and _1633_RLGPVQNEV_1641_ (pink trapezium) are also represented. Fixation events (mutations at frequency >90%) are represented with # in (e) and (f). The arcs shown in the inner circular area represent statistically significant cooccurring pairs of mutations that are shared by two or more viral variants. The asterisk (∗) in (e) denotes the potential compensatory mutation at position G1671R cooccurring with immune escape variants identified within _1602_RAQAPPPSW_1610_ (green).

We considered the evolutionary dynamics of circulating viral genomes and HCV specific CD8+ T-cell responses over the course of an infection.  Figure 3 shows the evolutionary dynamics of viral genomes in subject Ch_240 and the distribution of cooccurring genomic mutations and their relationship with viral diversity and T-cell responses.  Figure 3(a) shows the viral dynamics as already explained in Figure 2.  Figure 3(b) shows the experimental results (ELISPOT data) detailing the measurement of two HCV specific CD8+ T-cell responses targeting epitopes _1602_RAQAPPPSW_1610_ (green) and _1633_RLGPVQNEV_1641_ (pink) within the NS3 region. Immune responses are first detected at 44 DPI but at very low amount. In this context of early onset of CD8+ T-cell responses, and a concomitant genetic bottleneck event characterizing the circulating HCV viral population, we investigated the hypothesis that these immune responses were driving evolution of HCV genomes. We therefore assessed the distribution of mutations and their cooccurrence before and after this genetic bottleneck, as well as the evolution of these mutants in relation to the appearance of the two CD8+ T-cell responses identified within this region. Viral diversification was very limited within the first 57 DPI, and no statistically significant cooccurring mutations were observed. In particular, nonsynonymous substitution P1606S was observed within the CD8+ T-cell epitope _1602_RAQAPPPSW_1610_ at low frequency at 44 DPI but this mutation was not detected at 57 DPI, most possibly because it was at a frequency of occurrence lower than 1% (the minimum threshold detected after error correction of sequencing data). CD8+ T-cell responses against the two epitopes _1633_RLGPVQNEV_1641_ and _1602_RAQAPPPSW_1610_ were detected at increased magnitude at 85 DPI, close to the time of the first genetic bottleneck (Figure 3(a)). This was followed by the emergence of a new viral population after 100 DPI (Figure 2). Following this genetic bottleneck, a new mutation P1606L was identified within the epitope region _1602_RAQAPPPSW_1610_ (Figure 3(e)), which was shared among 80% of the viral population. This mutation is therefore likely to be an immune escape variant. Another mutation, G1671R, was then identified to cooccur with the immune escape mutation P1606L at 220 DPI. The mutation G1671R was not present when the P1606S was first identified, and these data suggest G1671R is potentially compensating for P1606L within the epitope region targeted by CD8+ T-cell responses. Secondly, P1606S occurred at a very low frequency at 44 DPI followed by nondetection in 57 DPI (perhaps due to extremely low frequency of occurrence), indicating the possibility that it is an individual deleterious mutation, which restricts the fitness or success of the escape variant. This analysis hence indicates that detection of cooccurring mutations over the course of the infection can be utilized to detect key patterns of immune escape.

As infection progressed, increased variability in the NS3 region at 538 DPI was observed (Figure 3(e)) approximately 300 days after the previous sequenced time point, despite the overall decline in viral diversity across the full genome (Figure 3(a)). This information suggests that the new viral population is evolving in a specific direction, where HCV viral variants possess mutations that enable the evasion of the CD8+ T-cell responses targeting the NS3 region. As observed in Figure 3(f), the paired mutations P1606L and G1671R that were detected at 220 DPI were lost at 538 DPI. New HCV genomic variants were identified at 538 DPI, with new mutations all cooccurring with P1606L, and a fixation event at V1641I. It is important to note that all HCV genomes at 538 DPI carry the epitope variant _1633_RLGPVQNEI_1641_. Closer examinations revealed that all cooccurring mutations were connected with either position P1606L, V1641I, or both. We also found a fixation event at position T1509N. However this was not a compensatory mutation as the same mutation was already fixed at 220 DPI.

### 3.4. Covariance Network Analysis

**Figure 4:**
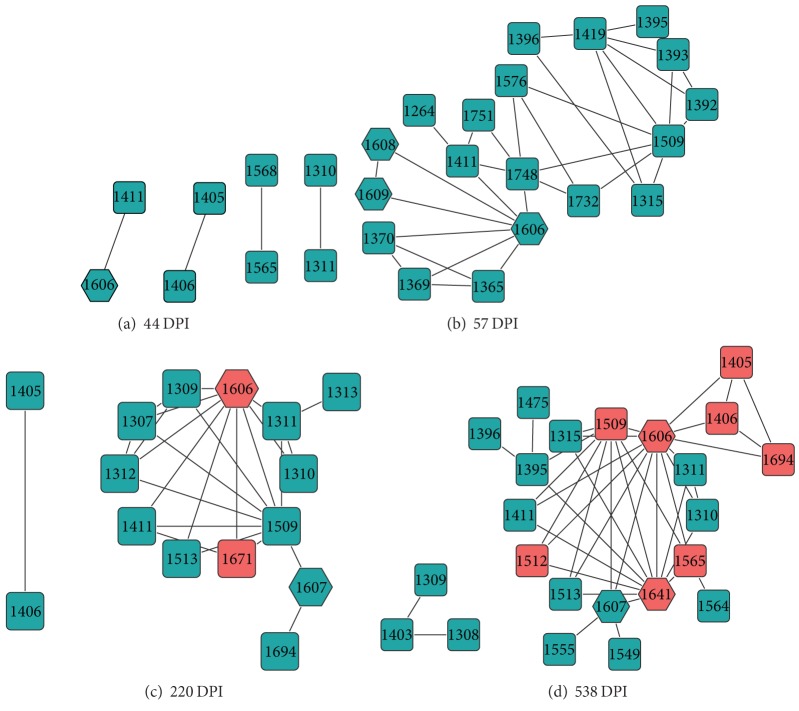
A representative example of the covariance network analysis. Network representation pair-wise covariance analysis of nonsynonymous mutations detected over the course of infection within the NS3 region (nucleotide region 4000–5499, amino acid region 1200–1800) in subject Ch_240. Nodes presented in the network are positions in the viral sequence found to have significant covariance values that indicate strong covariation between two mutation sites. In each network, the degree of connectivity is increasing from left to right. Hexagonal nodes denote mutation positions that lie within an epitope region, while those in red are mutations that have been detected as statistically significant cooccurring mutations using the Jaccard similarity coefficient. Covarying mutations, which are not shared between two or more viral variants, have their nodes colored in green.

We then analyzed, in a more general fashion, the network of genetic mutations that were identified during the course of the infection in subject Ch_240. To do so, we constructed a covariance score (see Section 2) between all the pairs spanning the entire region (amino acid region 1200–1800).  Figure 4 shows the network representing the evolving pattern of the connections between pairs of sites with mutations in the partial NS3 region of subject Ch_240. Each position with a mutation shown on the network is referred to as a node and each line drawn with a neighboring node is referred to as a connection. The most striking feature of the covariance network analysis is the evolving pattern of connections with node 1606. This node, which represents the mutations P1606S (44 DPI) and P1606L (220 DPI and 538 DPI), was observed in the epitope _1602_RAQAPPPSW_1610_ at the earliest time point and was subsequently detected in all other time points, with new connections at every time point.

Covariance network representing 44 DPI (Figure 4(a)) shows a small number of nodes with P1606S connecting to only one neighbor. Diversification of connections is seen at 57 DPI (Figure 4(b)) where the network is more complex due to an increase in the number of nodes and higher degree of connections between nodes. At 220 DPI (Figure 4(c)) an emerging pattern is revealed, where mutation P1606L appears to be a “hub” mutation, having connections with the majority of nodes within the network. 538 DPI (Figure 4(d)) shows a further increase in the number of new connections stemming from P1606L. In particular, the node corresponding to V1641I is another mutation that lies within the CD8+ T-cell epitope _1633_RLGPVQNEV_1641_. Close examination reveals that most nodes detected at 538 DPI (Figure 4(d)) are connected to P1606L or V1641I. These data suggest that the mutants P1606L and V1641I form a potential pair of mutations that is critical for viral escape dynamics.

### 3.5. Comparison of Cooccurring Mutation Analysis and Covariance Network Analysis

In the analysis of cooccurring pairs of genomic mutation (Figure 3) we showed that HCV immune escape dynamics are characterized by the existence of potential compensatory mutations, which characterize the HCV viral populations, thus suggesting that a strong adaptation mechanism is at play. The covariance network analysis shown in Figure 4 highlights the increasing complexity between the distributions of covarying mutations with the evolution of the infection. These data suggest that cooccurring mutations that affect immune response (hexagonal nodes in Figure 4) are also part of the set of genomic mutations that form the “hub” of the network of covarying mutations (see red nodes in Figure 4). For example, it is interesting to note how the covariance network shows an evolving and complex network of nodes, which supports the appearance of the G1671R being a compensatory mutation to P1606L at 220 DPI after the first genetic bottleneck, when increasing CD8+ T-cell responses were observed. Notably, the appearance of a novel viral population at 538 DPI characterized by the appearance of the pair P1606L and V1641I again reveals a new pattern of connected genetic mutations that contributes to the viral escape dynamics and eventually to viral chronic persistence in subject Ch_240. Interestingly, the network analysis between 44 and 220 DPI showed a rapid modification of the network of covarying mutations, which revealed unstable distribution of mutations connected to the mutation P1606L. This suggests that without the cooccurrence of G1671R at 220 DPI, the immune escape mutation P1606S carries a significant fitness cost, which may limit the survival capacity of this escape variant against CD8+ T-cell response. This is supported by the absence of P1606S at 57 DPI, where there may be a deleterious effect hindering the survivability of the variant.

At 538 DPI (Figure 4(d)), approximately 300 days after the previous time point, the covariance network highlights an increasing complexity of the network, where the majority of the nodes previously observed at 220 DPI disappear and only a few nodes are carried over. In particular, the mutation P1606L is characterized by a completely new set of covarying mutations, again confirming the results from the analysis of cooccurring mutation analysis. This analysis also revealed several highly connected genomic mutations that were also identified from the analysis of cooccurring mutation using the Jaccard similarity coefficient (red nodes in Figure 4). In particular, the fixation at position T1509N is also found as a highly connected node at 538 DPI. Moreover, this analysis identified a triplet of genetic mutations (K1405N, C1406S, and L1694R) that were also connected to the immune escape mutation P1606L, clearly indicating the existence of a subnetwork of evolving genomic variants.

### 3.6. Pipeline Validation with SGA Data

**Figure 5:**
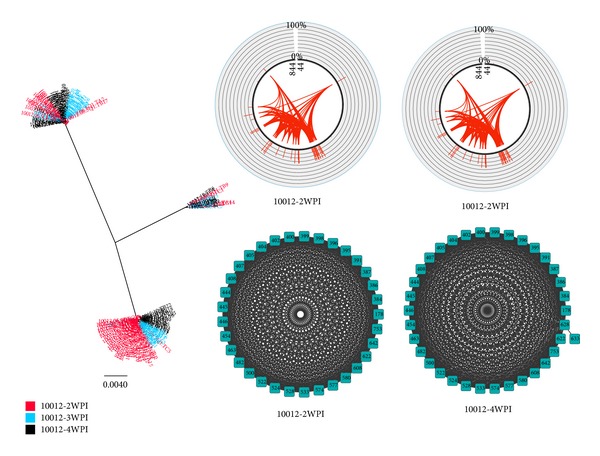
A representative example of a HCV infection presenting highly diverse viral population. Single Genome Amplification (SGA) data from longitudinally collected HCV populations over three time points (2, 3, and 4WPI) taken from a subject (10012) infected with HCV genotype 1a. This analysis was based on sequences from partial Core protein, p7, E1, and E2 protein of HCV from 2WPI and 4WPI (3WPI not shown). Unrooted phylogenetic tree displays three major subpopulations of viruses. A substantial viral diversification is observed since 2 weeks after infection. During this early stage of infection it is unlikely that HCV specific T-cell and B-cell responses are targeting the infection. However, the Circos plots highlight the presence of highly connected variants with specific patterns of cooccurring mutations. This is further validated from the covariance network showing high number of pairs of sites, which are maintained during the early phase of infection.

We validated the proposed bioinformatics pipeline with the analysis of sequences obtained from a subject infected with HCV (Sub 10012, data retrieved from Li et al. [22]) within the first month of infection (measured by weeks after infection or WPI). Given the early stage of infection, we hypothesize the absence of adaptive immune responses targeting those viral populations. Indeed, phylogenetic analysis of sequences from 5′ end to partial NS2 of the HCV genome showed the presence of a random evolution of three major viral populations arising from three transmitted/founder variants that successfully started the infection (Figure 5). The Circos plots show an overall increase in frequency of occurrence of these mutations over time, suggesting the presence of diversifying viral populations without immune pressure. However, despite this apparent random evolution, we identified several cooccurring mutations (Circos plots in Figure 5) that showed highly connected mutation patterns between these three viral populations. Therefore, there is evidence of inheritance of specific mutations throughout the viral evolution, with mutations that occur very early in infection being maintained during the generation of new variants. The covariance networks across the three time points (Figure 5) illustrate the positions where the inherited mutations occur. This highlights several pairs of genomic sites that mutate in each of these populations, thus representing hubs of the evolutionary dynamics of HCV genome during early infection. The comparison of subject 10012 and subject Ch_240 clearly indicates a very different evolutionary pattern driven by the presence of immune response.

## 4. Discussion

In analyzing viral sequences through the proposed pipeline, this study has revealed a pair of mutations within a region of the viral genome that may form the hub of a network of covarying mutations allowing viral persistence in subject Ch_240. Using the proposed bioinformatics pipeline we addressed the details of immune escape from longitudinal observations of viral evolution of HCV infection and provided insight into the evolution of the virus in relation to the selective pressure exerted by CD8+ T-cell immune responses. Moreover, this analysis provided evidence that the cooccurrence of P1606S and V1641I may be central to the success of immune escape variants against CD8+ T-cell responses targeting HCV during the establishment of chronic infection. We showed that HCV evolution under CD8+ T-cell response is characterized by a complex evolving pattern of mutations that consists of mutations in multiple regions functioning as a whole to provide the virus with the ability to escape immune pressure. This analysis, although limited to a small portion of the HCV, provides useful information in identifying potential factors that contributes to the virus's overall escape outcome.

In this study we presented a novel bioinformatics approach for the identification of key viral mutations events that dictate success of viral escape in the establishment of a chronic HCV infection. The output of our computational analysis offers a detailed description of the complex patterns characterizing immune escape dynamics during HCV infection and can therefore be relevant in studying immune escape dynamics in HCV and more in general in other rapidly mutating viruses. In combination with experimental data of CD8+ T-cell responses, our analysis provided a novel method to characterize the dynamics of compensatory mutations. As experimental measures, such as ELISPOT, only test CD8+ T-cell responses against specific epitopes of 8–10 amino acids, analysis of viral genomes is required to explore the distribution of other mutations outside the epitope region, which can serve as compensatory mutations. This analysis has remained elusive because of the lack of appropriate deep sequencing data to measure low frequency variants and the lack of appropriate methods to link distant mutations. With the rise of NGS technologies, and the development of new computational methods for haplotype reconstruction from NGS data [34], this information is becoming accessible. Hence the proposed bioinformatics pipeline is one of the first proposed to provide an exhaustive scenario of immune escape dynamics that takes both genomic and immunological data as input variables.

In the bioinformatics pipeline, we have also provided an array of graphical representation of evolving genomic mutations connected to each other. With the increasing availability of data and reduction of NGS cost, analyses using computational pipelines are necessary to unravel complexity of large data sets. Moreover, computational analyses can be utilized to minimize experimental costs and reduce time for manually laborious data processing. Since there is no direct monetary penalty in reruns of computational pipelines, exploratory procedures can be done on data with less limitation. For instance the pipeline could be used to obtain preliminary data on viral sequences prior to deciding whether certain regions of the HCV genome are worth spending resources, such as limited samples, or employing assays requiring a large number of cells to achieve sufficient specificity and sensitivity (e.g., ELISPOT or tetramer staining). Bioinformatics predictions of viral epitopes targeted by CD8+ T-cell responses are also available (see http://www.immuneepitope.org). These data can be substituted into experimental measurements and integrated in this pipeline.

The study performed on the partial NS3 region of Ch_240 is only one of many ways of using the pipeline. The tools designed in this pipeline are implemented specifically so that each component of the pipeline can be applied separately. Moreover, different types of viral sequences can be considered as input data. For example, haplotype reconstruction sequences from NGS data can be replaced with single genome amplification data, which are often used to study viral evolution [22] (see Figure 5). Furthermore, supporting tools in the package are able to take in generic fasta sequences and convert these into a format that can be processed in the pipeline.

There are a number of limitations in the proposed pipeline. For example, in this study, only pairs of cooccurring mutations were utilized, in order to limit the complexity of the results. Investigation of a higher number of cooccurring mutations (such as triplets or quadruplets) is possible. However as the length of the viral sequence under investigation increases, the number of cooccurring mutations will also increase (see for instance Figure 5). Using the pipeline without NGS data is also advantageous because current analysis suffers from highly error prone data. For instance, our analysis with haplotypes for subject Ch_240 was based on 454 Roche sequences data, which have been used to reconstruct viral haplotypes with computational demanding software packages. Although the viral genome reconstruction was successful in our study, this method still presents a high false positive rate when reconstructing low frequency variants [35, 36]. However, to address the issue, we have chosen to consider only viral variants reconstructed as haplotypes with a frequency of occurrence greater than 1%.

This computational and statistical framework can also be applied to other viruses and to identify more complex patterns of immune escape or drug resistance. For instance, understanding the dynamics of escape variants against both T and B cell responses. This is a common feature during infections with rapidly mutating viruses, such as HIV [37]. Moreover, the use of Jaccard coefficient allows the identification of specific patterns of mutations that are likely to cooccur more than random expectation. This could be for instance also applied to the detection of drug resistance mutations and for the identification of compensatory sites. The network of mutations performed with the covariance analysis holds a broader goal, and that is to screen viral sequences for major mutating sites or “hubs,” identifying sites that are mutating at a significantly high rate across the full genome.

## 5. Conclusion

This work proposed a novel bioinformatics pipeline for the analysis of immunological and virological data of viral infection, which simplifies the analysis and visualization of complex patterns of viral mutations during the course of an infection. It also allows for a statistical analysis of the relationship between viral mutations and the immune response targeting specific HCV variants. This type of software package is likely to become increasingly common in the near future, as a result of the increasingly large amount of data being rapidly generated and the overwhelming need for computational tools for analysis of complex multidisciplinary data in a time efficient manner.
